# Family resources and externalizing problem behaviors among “lads” in township junior high schools: the role of positive youth development and peers’ positive moral character

**DOI:** 10.3389/fpsyg.2026.1852822

**Published:** 2026-07-21

**Authors:** Shuyan Gao, Mengmeng Xie

**Affiliations:** 1 Students’ Mental Health Education Center, Mudanjiang Normal University, Mudanjiang, China; 2 Shandong Provincial Institute of Educational Sciences, Jinan, China

**Keywords:** externalizing problem behaviors, family resources, lads, peers’ positive moral character, positive youth development

## Abstract

**Background:**

Given the increasing occurrence of gang-like violent incidents in township junior high schools, investigating strategies for reducing the externalizing problem behaviors of “lads” (peer groups characterized by disruptive and delinquent tendencies) has emerged as a critical concern.

**Objectives:**

The purpose of this study was to examine the relationship between family resources and externalizing problem behaviors among “lads” in township junior high schools, the mediating effect of positive youth development, and the moderating effect of peers’ positive moral character.

**Methods:**

By convenient sampling, 608 “lads” from 15 township junior high schools in China completed the self-report questionnaires, including Family Resource Scale, Positive Youth Development Scale, Positive Moral Character Scale and Externalizing Problem Behavior Scale. A cross-sectional research design was conducted to test the moderated mediation model.

**Results:**

(1) Family resources negatively predict externalizing problem behaviors among “lads.” (2) Positive youth development mediates the relationship between family resources and externalizing problem behaviors among “lads.” (3) Peers’ positive moral character positively moderates the effects of family resources on both positive youth development and externalizing problem behaviors among “lads.” Higher levels of peers’ positive moral character strengthen the positive effect of family resources on positive youth development and the protective effect of family resources on externalizing problem behaviors.

**Conclusion:**

Both family resources and positive youth development serve as key protective factors against externalizing problem behaviors among “lads” in township junior high schools. The results indicate that educators can design interventions for these students based on positive youth development theory, which highlights the importance of resources and strengths.

## 1 Introduction

The term “lads” was introduced by the British social scientist Paul Willis to describe informal student groups with negative orientations. Such groups are typically characterized by common interests and close interpersonal ties; however, they often resist school rules and classroom management, interfere with classmates’ studies and daily lives, and may even engage in delinquent or criminal activities (Paul, 1978). During the research process, the authors found that “lads” exist in every township junior high school in China, accounting for one-fifth to one-quarter of the class. Compared to those in urban junior high schools, “lads” in township junior high schools are more active and widely recognized. In some cases, they may even join campus gangs, street corner youth gangs, or adult-organized criminal syndicates. They often make teachers and parents feel frustrated. Consequently, how to educate and guide them has become a major challenge.

Positioned at the margins of their classes and schools, “lads” are commonly identified by a series of externalizing problem behaviors. These behaviors are defined as observable maladaptive responses in which individuals or groups exhibit overtly confrontational or destructive actions toward others or their environment as a means of coping with inner distress or maladaptation due to poor self-control (Achenbach et al., 1987). Such behaviors include addictive behaviors, deceptive behaviors, disciplinary violations, aggressive behaviors, and delinquent acts (Xu et al., 2025; Lei et al., 2019; Fang et al., 2004). The externalizing problem behaviors of “lads” not only hinder their own physical and mental development but also adversely affect others, disrupt the teaching order of schools, and even threaten the social harmony. Moreover, compared to the externalizing problem behaviors of individuals, such behaviors of “lads” often unfold in the form of groups, thus are more destructive and harmful. With the frequent occurrence of gang-like violent incidents in township junior high schools in recent years, investigating the externalizing problem behaviors of “lads” has become an unavoidable issue. However, as of now, no relevant research has been conducted. Therefore, this study aims to reduce externalizing problem behaviors of “lads” and ultimately promote their positive transformation or de-grouping by exploring the mechanisms underlying such behaviors.

Previous studies have emphasized the role of risk factors in shaping the externalizing problem behaviors of adolescents, showing that reducing exposure to such risks can help mitigate these behaviors. However, focusing on positive factors offers a dual advantage: it not only reduces externalizing problem behaviors but also promotes the development of positive behaviors in adolescents. As these positive behaviors strengthen, they can gradually replace externalizing problem behaviors, thereby further reducing them to a greater extent (Chang et al., 2019). In recent years, with the prevalence of positive psychology research, the study and application of positive youth development theory have received increasing attention. According to positive youth development theory, the development of adolescents is plastic, and they can integrate various external resources to promote the development of individual strengths and potentials, thereby reducing externalizing problem behaviors (Lin et al., 2017). It can be seen that the positive youth development theory is a supplement and balance to the long-standing dominant “defect view,” and is in line with the essence of human nature and development. It provides a new and positive perspective for exploring the influencing factors and mechanisms of externalizing problem behaviors of “lads” in township junior high schools. Previous studies have found that positive factors can reduce externalizing problem behaviors among ordinary adolescents (Chang et al., 2019; Wang et al., 2022). It is still unknown whether they can also reduce externalizing problem behaviors among “lads” in township junior high schools. Therefore, from the perspective of positive youth development theory, this study examines how external developmental assets, such as family resources and peers’ positive moral character, along with internal developmental assets, specifically positive youth development, influence the externalizing problem behaviors of “lads” in township junior high schools.

## 2 Literature review

Developmental assets are a collection of experiences, resources, and opportunities that promote adolescents’ healthy and positive development while reducing the likelihood of problem and risk behaviors (Benson and Leffert, 2001). They can be considered as essential nutrients for adolescent growth. These assets are categorized as external and internal developmental assets. External developmental assets, also known as ecological resources, are environmental factors that promote adolescents’ healthy development. They mainly refer to the positive developmental experiences that adolescents obtain from others who strengthen connections and provide opportunities, such as family resources and peers’ positive moral character. Internal developmental assets refer to the value standards, competencies, and skills possessed by adolescents that guide their behaviors, such as positive youth development. This study investigates the relationship between family resources and externalizing problem behaviors of “lads” by examining the mediating effect of positive youth development and the moderating effect of peers’ positive moral character. The findings can provide a theoretical framework and empirical evidence to the practical work aimed at enhancing the physical and mental well-being of “lads” in township junior high schools

### 2.1 Family resources and externalizing problem behaviors

As one type of developmental assets, family resources refer to a series of experiences, relationships, skills, and values in the family context that effectively promote adolescents’ positive development (Search Institute, 2005). They include parental support, parent–child communication, family atmosphere, family norms, parental expectations and so on. High levels of family resources enable adolescents to receive adequate love and support from parents, experience a harmonious parent–child relationship, thrive in a nurturing home environment, and benefit from clear behavioral guidelines, effective supervision, and constructive parental guidance.

Ecosystem theory highlights the family as the most fundamental microsystem in the lives of adolescents, playing a crucial role in their behavioral development (Zhang et al., 2021). Empirical evidence has demonstrated that external developmental assets, such as family resources, serve as protective factors that help reduce externalizing problem behaviors in adolescents. Greater availability of positive family resources is associated with a stronger decrease in externalizing problem behaviors in adolescents (Chang et al., 2019). A latent profile study revealed that junior high school students with higher levels of family resources were less likely to exhibit internalizing and externalizing problem behaviors (Gai, 2021). In addition, family structure influences externalizing problem behaviors among rural adolescents. Rural adolescents from intact families are less likely to exhibit externalizing problem behaviors than those from incomplete families (Yang, 2023). A cross-lagged study found that parental warmth significantly and negatively predicted externalizing problem behaviors in migrant children, with warmer parenting associated with fewer such behaviors (Ma et al., 2021). Family functioning also significantly and negatively predicted the externalizing problem behaviors of adolescents. Good family functioning, such as love and behavioral discipline from parents, as well as positive parent–child communication, was shown to significantly reduce the likelihood of adolescents displaying externalizing problem behaviors (Wang et al., 2020). These findings highlight family resources as a key protective factor against externalizing problem behaviors of adolescents. Accordingly, we hypothesize that family resources predict the externalizing problem behaviors of “lads” in township junior high schools.

### 2.2 Mediating role of positive youth development

Positive youth development refers to the key attributes of healthy and fully developed adolescents, including character, competence, self-worth, and connectedness. These attributes reflect constructive, reciprocal interactions between individuals and their developmental environments (Lin et al., 2017). According to positive youth development theory, family resources are a core factor influencing adolescents’ positive development. Adolescents with more family resources tend to demonstrate stronger outcomes in positive youth development (Lv, 2022). International studies have found that a supportive family atmosphere is strongly and positively associated with various dimensions of positive youth development, fostering the growth of adolescents’ positive attributes (Ng and Sulaiman, 2017). Meta-analytical evidence indicated that positive parenting styles were positively correlated with enhanced outcomes in positive youth development (Tang et al., 2024). A retrospective interview study found that family environments that balance affection and rules, including democratic parenting styles, supportive parent–child relationships, appropriate parental demands and supervision, and positive parental role models, facilitate adolescents’ positive development (Lan and Gai, 2017). Cross-sectional studies have also demonstrated that family functioning (Liu et al., 2020) and parent–child relationships (Mai, 2023) are positive predictors of adolescents’ positive development. Consequently, it can be predicted that family resources predict the positive youth development of “lads” in township junior high schools.

Positive youth development is a key indicator of adolescent growth. Previous studies have consistently recognized it as a crucial internal developmental asset, highlighting its protective function in mitigating adolescents’ externalizing problem behaviors. Cross-sectional studies have demonstrated that positive youth development significantly and negatively predicts a range of externalizing problem behaviors, including substance abuse, deception, Internet addiction, running away from home, truancy, casual sexual activity, fighting, and theft (Sun and Shek, 2012; Chi and Cui, 2020; Dou and Shek, 2021). A longitudinal study found that positive youth development in the first year of junior high school significantly and negatively predicted externalizing problem behaviors in the second year (Wang et al., 2022). A latent profile analysis revealed that higher levels of positive youth development were correlated with fewer problem behaviors and stronger school adaptation among adolescents (Ye et al., 2017). An intervention study also reported that positive youth development programs significantly reduced students’ drug abuse and delinquent behaviors (Shek and Lu, 2011). Therefore, it can be predicted that positive youth development predicts the externalizing problem behaviors of “lads” in township junior high schools.

Based on the above analysis, we predict that positive youth development mediates the relationship between family resources and externalizing problem behaviors of “lads” in township junior high schools. More family resources may promote high levels of positive development, which leads to less externalizing problem behaviors among “lads.”

### 2.3 Moderating role of peers’ positive moral character

Positive moral character refers to moral qualities that are broadly recognized by society and contribute to individual well-being and social harmony (Liu and Meng, 2010). Peers’ positive moral character refers to the expression of these positive moral qualities among peers. During adolescence, students’ relationships with peers strengthen, and the focus of primary attachment shifts from parent–child relationships to peer relationships. Peer relationships become increasingly important and have a significant influence on adolescents’ attitudes and behaviors (Wang and Meng, 2024). Dependence on peers is particularly strong for “lads” in township junior high schools, and peers exert a greater influence on their behavior. Nevertheless, as minors, they continue to live primarily within the family environment, making them subject to the influence of family and peer contexts. From the perspective of ecosystem theory, different ecological environments interact and jointly influence individual development (Liu, 2021). Empirical studies have shown that the peer environment can moderate the relationship between family context and the psychological and behavioral development of adolescents. For instance, deviant peer affiliation moderated the relationship between parent–child attachment and psychological capital in junior high school students, with deviant peer affiliation weakening the positive influence of parent–child attachment on psychological capital (Wang et al., 2017). Peer acceptance moderated the relationship between parental psychological control and moral disengagement among vocational school students, with higher levels of peer acceptance reducing the impact of parental psychological control on moral disengagement (Liang and Liu, 2017). Peer relationships moderated the relationship between negative social environments, including negative family environments, and problem behaviors in left-behind and non-left-behind children, with supportive peer relationships mitigating the effects of negative social environments on children’s problem behaviors (Jin et al., 2012). Positive peer influence moderated the relationship between negative life events, including negative family life events, and problem behaviors among adolescents, with positive peer influence mitigating the effect of negative life events on problem behaviors (Jin and Zou, 2015). Thus, we predict that peers’ positive moral character moderates the relationship between family resources and both positive youth development and externalizing problem behaviors among “lads” in township junior high schools, with peers’ positive moral character strengthening the effect of family resources on both positive youth development and externalizing problem behaviors.

### 2.4 Research hypotheses

**Figure 1:**
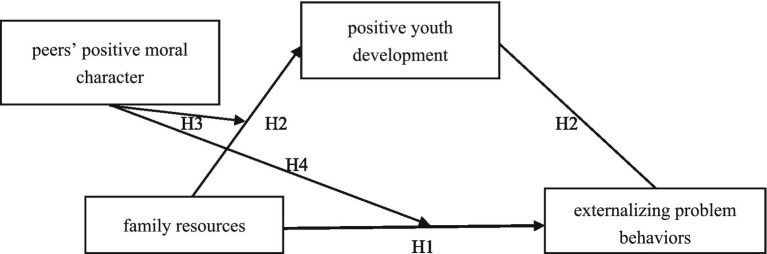
The moderated mediation model.

Based on the literature review, the following four hypotheses are proposed (Figure 1).

*Hypothesis 1*: Family resources predict the externalizing problem behaviors of “lads” in township junior high schools.

*Hypothesis 2*: Positive youth development mediates the relationship between family resources and externalizing problem behaviors of “lads” in township junior high schools.

*Hypothesis 3*: Peers’ positive moral character moderates the relationship between family resources and positive youth development of “lads” in township junior high schools. Higher levels of peers’ positive moral character strengthen the positive effect of family resources on positive youth development.

*Hypothesis 4*: Peers’ positive moral character moderates the relationship between family resources and externalizing problem behaviors of “lads” in township junior high schools. Higher levels of peers’ positive moral character strengthen the protective effect of family resources on externalizing problem behaviors.

## 3 Methods

### 3.1 Participants

According to the requirements for sample size in structural equation modeling (Kline, 2023), a sample size of ≥200 is considered “moderate,” while a sample size of ≥400 is considered “good.” Adopting convenient sampling methods, a total of 92 groups of “lads,” comprising 627 students, were selected from 15 township junior high schools in Heilongjiang Province of China. The participants were required to meet three criteria: (1) they must be currently enrolled students in township junior high schools with registered school records. (2) they must fit the definition of “lads”: a group of students who share common interests, maintain close interpersonal ties, resist school rules and classroom management, interfere with classmates’ studies and daily lives, and may even engage in delinquent or criminal activities. (3) they must be evenly distributed in terms of demographic characteristics such as gender and grade. Questionnaires were administered, yielding 608 valid responses, for a response rate of 96.97%. The mean age of the participants was 15.03 years (SD = 0.62). The remaining basic information is presented in Table 1.

**Table 1:** Basic information of the subjects.

Variable	Category	*n*	%
Gender	Male	351	57.73
	Female	257	42.27
Grade	7	193	31.74
	8	282	48.38
	9	133	21.88

### 3.2 Measures

#### 3.2.1 Family resource scale

The Family Resources Subscale of the Developmental Assets Scale (Chinese version) developed by the Search Institute (2005) was used. This subscale includes 10 items, such as “I seek my parents’ opinions” and “I feel safe at home.” A 4-point scoring system ranging from 1 (never) to 4 (always) is used. The total score is calculated by summing the responses to all 10 items, with possible total scores ranging from 10 to 40. Higher total scores indicate higher levels of family resources. In this study, the scale demonstrated good reliability, with a Cronbach’s *α* of 0.94.

#### 3.2.2 Positive youth development scale

The *Chinese Positive Youth Development Scale (Short Form)* developed by Chai et al. (2020) was used. This scale comprises 48 items across four dimensions: character, competence, self-worth, and connectedness, including items such as “I understand the hardship of my parents” and “I have strong self-control.” A 5-point scoring system ranging from 1 (not true at all) to 5 (completely true) is used. The total score is calculated by summing the responses to all 48 items, with possible total scores ranging from 48 to 240. Higher total scores reflect higher levels of positive youth development. In this study, the scale demonstrated good reliability, with a Cronbach’s *α* of 0.98.

#### 3.2.3 Positive moral character scale

The *Assessment Scale of Positive Moral Character for Middle School Students* developed by Liu and Meng (2010) was used. This scale comprises 68 items across six dimensions: trustworthiness, respect, responsibility, fairness, caring, and civic duty, including items such as “Peers will do the things which they say they will do” and “If peers fail to do something, he/she will admit it truthfully.” A 5-point scoring system ranging from 1 (never) to 5 (always) is used. The scale includes 30 reverse-scored items. After reverse-scoring these items, the total score is calculated by summing the responses to all 68 items, with possible total scores ranging from 68 to 340. Higher total scores indicate higher levels of positive moral character in peers. In this study, the scale demonstrated good reliability, with a Cronbach’s *α* of 0.97.

#### 3.2.4 Externalizing problem behavior scale

The *Adolescent Externalizing Problem Behavior Scale* developed by Wang et al. (2010) was used. This scale includes 16 items, such as “fighting” and “skipping school or being truant.” A 5-point scoring system ranging from 1 (0 times) to 5 (≥6 times) is used. The total score is calculated by summing the responses to all 16 items, with possible total scores ranging from 16 to 80. Higher total scores indicate more frequent engagement in externalizing problem behaviors. In this study, the scale demonstrated good reliability, with a Cronbach’s α of 0.97.

### 3.3 Procedure

The current study was approved by the Ethics Committee of Mudanjiang Normal University. Prior to data collection, written informed consent was obtained from parents or legal guardians of all participants, and assent was obtained from the participants themselves. In addition, permission was secured from the principals of the 15 township junior high schools. Then the first author entered the classroom as a researcher. The participants filled out the questionnaires in the classroom accompanied by their head teachers. Participants were informed of their right to refuse participation and to withdraw at any time without any negative consequences.

### 3.4 Data analysis

We adopted a cross-sectional research design to test the hypothesized moderated mediation model. Data analysis was conducted using SPSS 26.0 and Mplus 8.3. Common method bias tests and correlation analyses were performed in SPSS 26.0, while structural equation modeling and hypothesis testing were conducted in Mplus 8.3.

## 4 Results

### 4.1 Common method bias test

Harman’s single-factor test was conducted. The analysis identified 21 factors with eigenvalues greater than 1, and the first factor accounted for 32% of the variance, which is below the critical value of 40%. These results indicate that there is no significant common method bias in this study.

### 4.2 Correlation analysis

Pearson correlation analyses were conducted for all study variables after controlling for gender and grade. The results revealed that family resources, peers’ positive moral character, and positive youth development were all significantly positively correlated with one another, and each was significantly negatively correlated with externalizing problem behaviors (see Table 2).

**Table 2:** Results of descriptive statistics and correlation analysis of variables (*n* = 608).

Variables	M	SD	1	2	3	4
1. Family resources	1.35	0.87	—			
2. Peers’ positive moral character	2.78	0.66	0.49**	—		
3. Positive youth development	2.00	0.71	0.52**	0.50**	—	
4. Externalizing problem behaviors	4.39	0.87	−0.55**	−0.42**	−0.50**	—

**p < 0.01, following the conventional thresholds recommended by Cohen (1994) and the American Psychological Association (2020).

### 4.3 Structural equation modeling and hypothesis testing

Structural equation modeling (SEM) was conducted using Mplus 8.3. First, confirmatory factor analysis was performed to assess the measurement model before examining the moderated mediation effect. The results indicated a good model fit, with *χ*^2^/df = 1.97, CFI = 0.90, TLI = 0.90, RMSEA = 0.04, and SRMR = 0.05. Next, the Latent Moderated Structural Equations (LMS) method proposed by Fang and Wen (2018) was employed to construct the SEM to test the moderated mediation effect. The analysis of the moderated mediation SEM followed a fixed three-step procedure.

Step 1: Assess the acceptability of the baseline SEM model without latent moderation terms. If the model is acceptable, proceed to Step 2; if not, the analysis is terminated. In this study, the baseline model demonstrated good fit, with *χ*^2^/df = 3.37, CFI = 0.90, TLI = 0.90, RMSEA = 0.06, and SRMR = 0.08, allowing the analysis to proceed to Step 2.

Step 2: Evaluate the acceptability of the moderated mediation SEM model that includes latent moderation terms. If the model is acceptable, proceed to Step 3; if not, the analysis is terminated. In this study, the moderated mediation SEM model showed an AIC of 100367.01, a decrease of 65.58 compared to the AIC of the baseline SEM model (100432.59), indicating an improved fit. The Log Likelihood of the moderated mediation SEM model was −49928.51, an increase of 34.79 compared to the baseline SEM model (−49963.30). The -2LL value was 69.58 with 2 additional degrees of freedom, and the chi-square test for -2LL was significant (*p* < 0.001). These findings indicate that the moderated mediation SEM model outperformed the baseline SEM model, allowing the analysis to proceed to Step 3.

Step 3: The product-of-coefficients method was employed to examine the moderated mediation effect. A moderated mediation effect is considered significant if the bootstrap confidence interval does not include 0. In this study, the results indicated that the index of moderated mediation was −0.04, and the 95% confidence interval was [−0.08, −0.01], excluding 0. According to the product-of-coefficients method, the mediating effect of family resources on the externalizing problem behaviors of “lads” through positive youth development was moderated by peers’ positive moral character. Specifically, peers’ positive moral character moderated the first half and direct paths of the mediated model. In other words, peers’ positive moral character moderated the effects of family resources both on positive youth development and externalizing problem behaviors of “lads.” The detailed results are presented below.

#### 4.3.1 Mediation effect test

**Figure 2:**
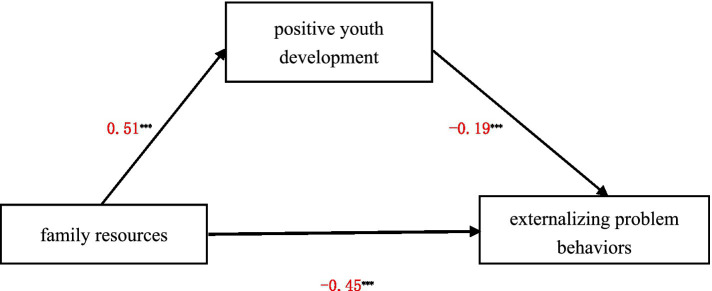
The mediation effect model. ***p < 0.001.

After controlling for gender and grade, a structural equation model was constructed with family resources as the independent variable, positive youth development as the mediator, and externalizing problem behaviors as the dependent variable to test the mediation effect. As illustrated in Figure 2, family resources significantly and negatively predicted externalizing problem behaviors (*β* = −0.45, *p* < 0.001, 95% CI [−0.63, −0.41]), supporting Hypothesis 1. In addition, family resources significantly and positively predicted positive youth development (*β* = 0.51, *p* < 0.001, 95% CI [0.36, 0.57]), and positive youth development significantly and negatively predicted externalizing problem behaviors (*β* = −0.19, *p* < 0.001, 95% CI [−0.34, −0.14]). These findings indicate that positive youth development may mediate the relationship between family resources and externalizing problem behaviors. The significance of the mediation effect was further examined using the bias-corrected bootstrap method with 5,000 resamples. As presented in Table 3, the mediating pathway “family resources → positive youth development → externalizing problem behaviors” showed a mediation effect of −0.11, accounting for 17.46% of the total effect (*p* < 0.001, 95% CI [−0.18, −0.06]). These results indicate that positive youth development mediates the relationship between family resources and externalizing problem behaviors of “lads” in township junior high schools, supporting Hypothesis 2.

**Table 3:** Results of mediation effect value and bootstrap test.

Effect	Path	Value	Boot LLCI	Boot ULCI	Proportion
Direct effect	Family resources → externalizing problem behaviors	−0.52	−0.63	−0.41	82.54%
Indirect effect	Family resources → positive youth development → externalizing problem behaviors	−0.11	−0.18	−0.06	17.46%
Total effect		−0.63	−0.74	−0.53	100%

#### 4.3.2 Moderation effect test

**Figure 3:**
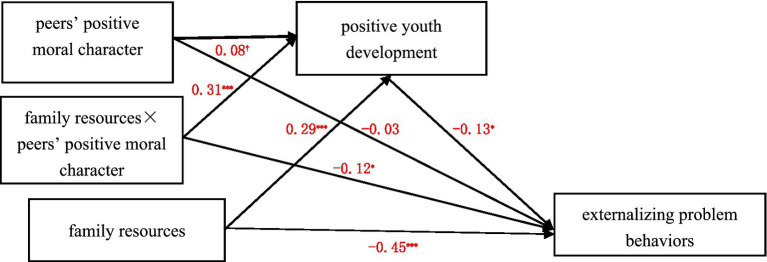
The moderated mediation effect model. †*p* < 0.10, *p < 0.05, ***p < 0.001.

After incorporating the interaction term into the mediation SEM, the results (see Figure 3) indicated that family resources significantly and positively predicted positive youth development (*β* = 0.29, *p* < 0.001, 95% CI [0.21, 0.36]). In addition, the interaction between family resources and peers’ positive moral character significantly and positively predicted positive youth development (*β* = 0.31, *p* < 0.001, 95% CI [0.24, 0.39]). These findings reveal that peers’ positive moral character positively moderates the relationship between family resources and positive youth development of “lads” in township junior high schools, supporting Hypothesis 3. Family resources significantly and negatively predicted externalizing problem behaviors (*β* = −0.45, *p* < 0.001, 95% CI [−0.54, −0.36]). In addition, the interaction between family resources and peers’ positive moral character significantly and negatively predicted externalizing problem behaviors (*β* = −0.12, *p* < 0.05, 95% CI [−0.22, −0.03]). These results indicate that peers’ positive moral character positively moderates the relationship between family resources and externalizing problem behaviors of “lads” in township junior high schools, supporting Hypothesis 4.

**Figure 4:**
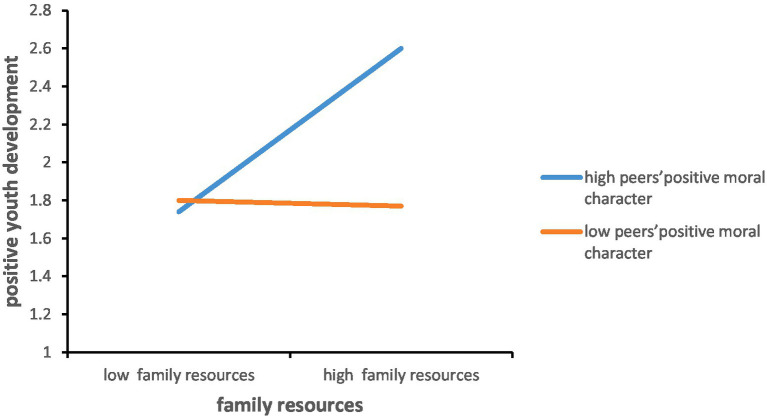
The moderating effect of peers’ positive moral character on the relationship between family resources and positive youth development.

**Figure 5:**
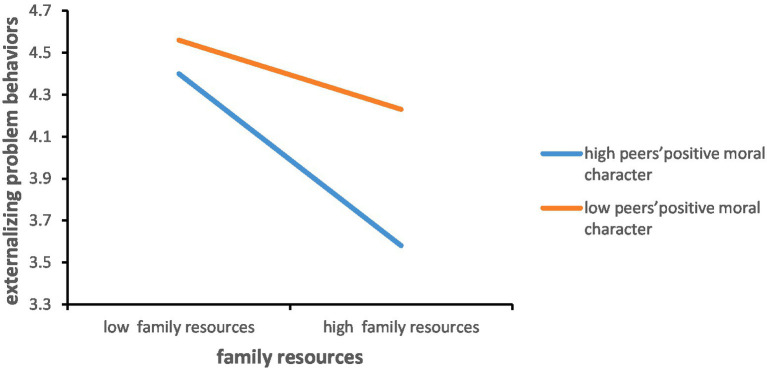
The moderating effect of peers’ positive moral character on the relationship between family resources and externalizing problem behaviors.

To further examine the moderation effect, the peers’ positive moral character was categorized into high and low groups using one standard deviation above and below the mean, and simple slope analyses were performed. The results indicated that the effect of family resources on positive youth development was not significant when peers’ positive moral character was low (M − 1SD) (*β* = 0.03, *p* > 0.05). When peers’ positive moral character was high (M + 1SD), family resources significantly and positively predicted positive youth development (*β* = 0.60, *p* < 0.001). This indicates that peers’ positive moral character enhances the influence of family resources on positive youth development of “lads,” such that higher levels of peers’ positive moral character amplify the effect of family resources on enhancing positive youth development (Figure 4). Similarly, simple slope analyses for externalizing problem behaviors showed that when peers’ positive moral character was low (M − 1SD), family resources significantly and negatively predicted externalizing problem behaviors (*β* = −0.33, *p* < 0.001). When peers’ positive moral character was high (M + 1SD), family resources still significantly and negatively predicted externalizing problem behaviors (*β* = −0.57, *p* < 0.001), with a steeper slope. This indicates that peers’ positive moral character enhances the effect of family resources on reducing externalizing problem behaviors of “lads,” such that higher levels of peers’ positive moral character increase the impact of family resources in decreasing externalizing problem behaviors (Figure 5).

## 5 Discussion

### 5.1 Impact of family resources on externalizing problem behaviors

This study revealed a negative relationship between family resources and externalizing problem behaviors of “lads” in township junior high schools. Family resources significantly and negatively predicted these behaviors, indicating that higher levels of family resources are associated with a lower likelihood of externalizing problem behaviors. This result is consistent with a previous research indicating that students who have more family resources are less likely to exhibit externalizing problem behaviors (Gai, 2021). Thus, family resources act as an important external protective factor that influences the externalizing problem behaviors of “lads.”

The family is the primary support system for “lads.” High levels of family resources reflect a complete family structure, a positive family atmosphere, and positive parenting styles, enabling “lads” to experience positive communication, affirmation, and care within the family. Family support and guidance enable “lads” to leverage family resources to address problems, exercise self-control, and adhere to social rules, thereby reducing externalizing problem behaviors. High levels of family resources also reflect parents’ ability to establish behavioral expectations, offer guidance, set clear norms, and monitor behaviors of “lads,” further decreasing the occurrence of externalizing problem behaviors. In addition, based on self-determination theory, “lads” with abundant family resources can satisfy their needs for autonomy, relatedness, and competence within the family context. Meeting these basic psychological needs fosters the development of prosocial behaviors and lowers the risk of externalizing problem behaviors (Ryan and Deci, 2017). Consequently, family resources play a critical role in mitigating externalizing problem behaviors among “lads.”

### 5.2 Mediating role of positive youth development

This study revealed that positive youth development partially mediates the relationship between family resources and externalizing problem behaviors among “lads.” Specifically, family resources exert a direct negative effect on externalizing problem behaviors and an indirect negative effect on these behaviors through positive youth development, supporting the theory of positive youth development. According to this theory, character, competence, self-worth, and connectedness represent key internal developmental assets that link external developmental assets to behavioral outcomes (Lerner et al., 2015). Previous studies have rarely explored the relationship between family resources, positive youth development, and externalizing problem behaviors from an integrated perspective. However, there have been explorations from a certain aspect or single dimension, such as that family function negatively predicts adolescents’ externalizing problem behavior through resilience (Wang et al., 2020), and that the positive parenting styles negatively predict children’s externalizing problem behaviors through behavioral regulation (Marcone et al., 2020), all of which are similar to the result of this study.

On one hand, family resources positively predict positive youth development. High levels of family resources reflect a complete family structure, a positive family atmosphere, and positive parenting styles, allowing “lads” to receive high-quality companionship, sufficient care, and scientific discipline. These factors foster the development of character, competence, self-worth, and connectedness (Xu, 2017; Jiang and Zhang, 2020; Li and Li, 2025; Li et al., 2018). On the other hand, positive youth development negatively predicts externalizing problem behaviors. According to the theory of developmental assets, high levels of positive youth development allow individuals to effectively manage the relationship between personal strengths and environmental resources, protecting them from external stressors and promoting adaptive development, which in turn reduces the likelihood of externalizing problem behaviors (Lerner et al., 2006). For “lads,” high levels of positive youth development enhance their internal self-regulation and enable the effective use of internal and external developmental assets to cope with real-life challenges in a constructive way, ultimately decreasing externalizing problem behaviors. It can be seen that high levels of family resources promote the positive development of “lads,” thereby reducing their externalizing problem behaviors. Thus, as an internal developmental asset, positive youth development functions as a crucial protective factor against externalizing problem behaviors of “lads.”

### 5.3 Moderating role of peers’ positive moral character

#### 5.3.1 Moderating role between family resources and positive youth development

This study demonstrated that peers’ positive moral character positively moderates the relationship between family resources and positive youth development among “lads.” Although high levels of family resources support positive youth development, the presence of peers with high positive moral character enhances the effectiveness of these resources, leading to even higher levels of positive youth development.

Specifically, when peers exhibit high levels of positive moral character, “lads” are influenced by their peers, which strengthens their own positive moral character. This influence promotes the development of moral cognition, moral emotions, moral will, and moral behavior, fostering positive patterns of thought, emotion, and action. Thus, “lads” can recognize themselves, evaluate others, and adapt to the society constructively and positively, thereby making full use of external developmental assets, such as family resources, to foster their positive development (Snyder and Lopez, 2013). Consequently, higher levels of peers’ positive moral character strengthen the positive effect of family resources on positive youth development. Conversely, when peers exhibit low levels of positive moral character, “lads” are influenced by peers who display poor moral character. This can diminish their moral cognition, moral emotion, moral will, and moral behavior, fostering a generally passive and negative orientation. Thus, “lads” are less able to leverage external developmental assets, such as family resources, which hinders positive development. Consequently, lower levels of peers’ positive moral character reduce the positive effect of family resources on positive youth development. These findings indicate that peers’ positive moral character, as an external developmental asset, amplifies the beneficial effects of family resources on positive youth development, supporting the “dual-protective factor” model, which points that one protective factor can strengthen the positive effect of another protective factor on positive outcomes (Yang et al., 2019).

#### 5.3.2 Moderating role between family resources and externalizing problem behaviors

This study revealed that peers’ positive moral character positively moderates the negative relationship between family resources and externalizing problem behaviors among “lads.” While high levels of family resources help reduce externalizing problem behaviors, the presence of peers with high positive moral character enhances the impact of family resources, further decreasing the occurrence of externalizing problem behaviors. Consistent with ecological systems theory, which posits that ecological subsystems jointly influence adolescents’ behavioral development rather than acting independently (Liang and Liu, 2017), this study shows that family and peer microsystems interact to influence externalizing problem behaviors of “lads.”

Specifically, when peers exhibit high positive moral character, “lads” are influenced by their peers, strengthening their own positive moral character. This promotes the development of mature personality traits of “lads” (Shen and Shen, 2018). Therefore, “lads” are more likely to respond positively to parental supervision, appreciate a harmonious family environment, and embrace positive parenting styles. Consequently, the role and influence of the family are strengthened, allowing positive parental guidance to be received and internalized, which decreases the likelihood of externalizing problem behaviors. Thus, higher levels of peers’ positive moral character strengthen the protective effect of family resources on externalizing problem behaviors. Conversely, when peers exhibit low levels of positive moral character or display more negative moral traits, “lads” may be influenced to adopt similar behaviors, which can hinder overall personality development and reduce responsiveness to parental supervision, harmonious family environment, and positive parenting styles. Thus, the benefits of high levels of family resources are not fully realized, increasing the likelihood of externalizing problem behaviors. Therefore, low levels of peers’ positive moral character decrease the protective effect of family resources on externalizing problem behaviors.

It can be seen that although family resources provide a protective effect against externalizing problem behaviors, low levels of peers’ positive moral character or the presence of negative moral traits can partially undermine this effect, leading to an increase in such behaviors. Therefore, peers’ positive moral character warrants significant attention. Efforts to reduce externalizing problem behaviors among “lads” should focus on peers’ influences, particularly on peers with lower levels of positive moral character. Implementing moral education can help enhance the positive moral character of peers and strengthen its protective role.

### 5.4 Limitations

This study has three primary limitations. First, the generalizability of the findings should be interpreted with caution. The sample was limited to “lads” from township junior high schools in Heilongjiang Province of China, and participants from other regions were excluded. Therefore, the findings may not be generalized to all “lads” in township junior high schools. To increase the representativeness, future research should include samples from multiple provinces and regions. Second, the use of self-report questionnaires may have introduced social desirability bias. To present themselves in a more favorable light, “lads” may have underreported their externalizing problem behaviors or overreported their positive youth development, which may limit the accuracy of the findings. Future research should combine self-report and other-report measures to provide a more comprehensive assessment of each variable. Third, causal relationships cannot be inferred from this study. As a cross-sectional study, cause-and-effect relationships among the variables cannot be established. For instance, while we interpret family resources as a predictor of positive youth development, the reverse direction (positive youth development enhancing family resources) is also theoretically plausible. Future research should utilize longitudinal or experimental designs to investigate potential causal relationships.

### 5.5 Educational implications

Previous studies have primarily focused on ordinary adolescents’ externalizing problem behaviors, but less attention has been given to “lads” in township junior high schools and their externalizing problem behaviors. Meanwhile, previous related studies lack positive research perspectives and scientific research paradigms, which make the reference and generalizability of conclusions questionable. However, this study has made breakthroughs in all of the above. Therefore, this study is innovative and can be regarded as a pioneering study in China.

The findings of this study indicate three practical strategies for reducing externalizing problem behaviors among “lads” in township junior high schools. First, parents should ensure the availability of adequate and effective family resources, including maintaining a stable family structure, creating a harmonious family atmosphere, adopting positive parenting styles and so on. Second, schools should promote positive youth development of “lads” through comprehensive positive education curriculums which can be implemented in three stages: providing positive education training for teachers and parents, delivering positive education curriculums for “lads,” and extending related extracurricular activities (Zeng and Zhao, 2018). Third, schools should foster positive moral character of peers through moral education which can be achieved in three ways: stimulating positive emotions, improving self-efficacy, and applying the appreciative inquiry approach among peers (Zhou, 2014). Family and school education should work in close coordination, with school education taking the lead, to collectively reduce externalizing problem behaviors among “lads” in township junior high schools.

In addition, this study finds that positive youth development plays a mediating role between family resources and externalizing problem behaviors, suggesting that we can reduce the externalizing problem behaviors of “lads” by implementing positive youth development programs. Foreign programs such as the 4-H intervention program, social emotional learning program, Miami youth development program, and community care program, as well as domestic programs such as the PATH program in Hong Kong and the psychological resilience intervention program in mainland China, can all be used for reference. These programs focus on both cultivating personal strengths such as character, competence, self-worth, and connectedness, and constructing supportive external environments. More importantly, they emphasize promoting the interaction between personal strengths and supportive external environments, thereby achieving the goal of reducing adolescents’ externalizing problem behaviors (Lin, 2023). Consequently, positive youth development programs can be used to reduce the externalizing problem behaviors of “lads” in township junior high schools.

## 6 Conclusion

The conclusions of this study are summarized as follows:

1. Family resources negatively predict externalizing problem behaviors of “lads” in township junior high schools.
2. Positive youth development mediates the relationship between family resources and externalizing problem behaviors of “lads” in township junior high schools.
3. Peers’ positive moral character positively moderates the effects of family resources on both positive youth development and externalizing problem behaviors of “lads” in township junior high schools. Specifically, higher levels of peers’ positive moral character strengthen the positive effect of family resources on positive youth development and the protective effect of family resources on externalizing problem behaviors.

## Data Availability

The datasets presented in this study can be found in online repositories. The names of the repository/repositories and accession number(s) can be found in the article/supplementary material.
